# Lead, Cadmium and Zinc Phytotoxicity Alter DNA Methylation Levels to Confer Heavy Metal Tolerance in Wheat

**DOI:** 10.3390/ijms20194676

**Published:** 2019-09-20

**Authors:** Sarfraz Shafiq, Qudsia Zeb, Asim Ali, Yasar Sajjad, Rashid Nazir, Emilie Widemann, Liangyu Liu

**Affiliations:** 1 Department of Anatomy and Cell Biology, University of Western Ontario, 1151 Richmond St, London, ON N6A5B8, Canada; 2 Department of Environmental Sciences, COMSATS University Islamabad, Abbottabad campus, Pakhtunkhwa 22060, Pakistan; asim21pk@hotmail.com (A.A.); rashidnazir@ciit.net.pk (R.N.); 3 College of Life Sciences, Capital Normal University, Beijing 100084, China; zeb_qudsia@yahoo.com; 4 Department of Biotechnology, COMSATS University Islamabad, Abbottabad campus, Pakhtunkhwa 22060, Pakistan; yasarsajjad@ciit.net.pk; 5 Department of Biology, University of Western Ontario, 1151 Richmond St, London, Ontario, N6A5B8, Canada; ewidema4@uwo.ca

**Keywords:** DNA methylation, ABCC transporters, HMA2, wheat, metal stress tolerance

## Abstract

Being a staple food, wheat (*Triticum aestivum*) nutritionally fulfills all requirements of human health and also serves as a significant link in the food chain for the ingestion of pollutants by humans and animals. Therefore, the presence of the heavy metals such as lead (Pb) and cadmium (Cd) in soil is not only responsible for the reduction of wheat crop yield but also the potential threat for human and animal health. However, the link between DNA methylation and heavy metal stress tolerance in wheat has not been investigated yet. In this study, eight high yielding wheat varieties were screened based on their phenotype in response to Pb stress. Out of these, Pirsabak 2004 and Fakhar-e-sarhad were identified as Pb resistant and sensitive varieties, respectively. In addition, Pirsabak 2004 and Fakhar-e-sarhad varieties were also found resistant and sensitive to Cd and Zinc (Zn) stress, respectively. Antioxidant activity was decreased in Fakhar-e-sarhad compared with control in response to Pb/Cd/Zn stresses, but Fakhar-e-sarhad and Pirsabak 2004 accumulated similar levels of Pb, Cd and Zn in their roots. The expression of *Heavy Metal ATPase 2 (TaHMA2)* and *ATP-Binding Cassette (TaABCC2/3/4)* metal detoxification transporters are significantly upregulated in Pirsabak 2004 compared with Fakhar-e-sarhad and non-treated controls in response to Pb, Cd and Zn metal stresses. Consistent with upregulation of metal detoxification transporters, CG DNA hypomethylation was also found at the promoter region of these transporters in Pirsabak 2004 compared with Fakhar-e-sarhad and non-treated control, which indicates that DNA methylation regulates the expression of metal detoxification transporters to confer resistance against metal toxicity in wheat. This study recommends the farmers to cultivate Pirsabak 2004 variety in metal contaminated soils and also highlights that DNA methylation is associated with metal stress tolerance in wheat.

## 1. Introduction

Plants encounter many environmental stresses during their life cycles and have consequently developed the ability to combat those variations that adversely affect growth, development and reproduction. Among them, heavy metal stress affects the fitness, survival and yield of crop plants during the course of their development by impairing the molecular, biochemical and physiological processes [1]. The heavy metals, lead (Pb) and cadmium (Cd) are highly toxic trace pollutants for humans, animals and plants [2,3]. Pb exposure to plants results in impaired root growth and germination, alterations in membrane permeability, water regime, hormonal status, disarrays in mineral nutrition, decrease in photosynthesis, transpiration, DNA synthesis and increased generation of reactive oxygen species (ROS) [2,4]. Similar to Pb, Cd toxicity is also associated with impaired plant growth, development, metabolism, enzyme activities, etc. [5,6]. In contrast to these toxic metals, essential metals like zinc (Zn) and iron (Fe) are needed for plants during their development in order to perform their vital physiological and biochemical functions [7,8]. Therefore, Zn deficiency is associated with impaired plant growth, yield and grain quality [9]. However, excess of Zn may also cause toxicity and affect the plant physiology [9]. Therefore, uptake, storage and utilization of these heavy metals are tightly controlled in plants to maintain their concentration in different cellular compartments.

To cope with heavy metals, plants have evolved either avoidance of uptake or tolerant mechanisms, including detoxification. The detoxification of heavy metals is associated with the exclusion of heavy metals from the cells, phytochelatin synthesis, sequestration of heavy metals into the vacuoles, binding to glutathione and amino acids [10]. The multidrug resistance-associated proteins (MRPs) belong to a subclass of ATP-binding cassette (ABC) transporters and are involved in heavy metal detoxifications, vacuolar sequestration of metabolites, pathogen response and plant development in *Arabidopsis* (*Arabidopsis thaliana*) [11,12,13,14]. Similar to *Arabidopsis*, ABCC-MRP from yeast and human have been reported to play a role in metal detoxifications [15,16], suggesting a conserved mechanism of ABCC-MRP transporters among different organisms. Wheat (*Triticum aestivum*) contains 18 *ABCC-MRP* genes [17] and *TaABCC3* has earlier been reported to play a role in grain development and resistance against secreted mycotoxin from *Fusarium* [18]. Another *ABCC-MRP* partial gene has been suggested to play a role in xenobiotic detoxification in wheat [19], indicating the important function of TaABCCs in wheat plant resistance.

In parallel to ABCC-MRP, plants have also evolved another system to prevent a cytotoxic concentration by effluxing the metals from the cytosol to the apoplast through the action of heavy metal ATPases (HMAs), also known as PIB type-ATPases. HMAs have been reported to play a role in heavy metal tolerance in *Arabidopsis* [20], and are well-conserved proteins among different organisms [21]. HMAs are associated with the transport of Zn, Cd, cobalt (Co), Pb, copper (Cu) and silver (Ag) [22,23]. The heavy metal ATPase2, TaHMA2, is a plasma membrane transporter from wheat that was suggested to export the Zn/Cd toward the apoplast [24]. Yeast expressing wheat *TaHMA2* was found resistant to Zn/Cd and the over expression of *TaHMA2* conferred a mild resistance against Zn and Cd in *Arabidopsis*, suggesting the important function of TaHMA2 in metal tolerance in wheat.

Chromatin landscape becomes dynamic in response to environmental and developmental cues, thus modulates the DNA accessibility to regulate gene expression and thus controls the various cellular and physiological processes [25]. DNA methylation is involved in various biological processes including flowering time, imprinting, flower and leaf morphogenesis, fertility through gene silencing [26,27]. Different DNA methyltransferases are involved in DNA cytosine methylation of three different sequence contexts, i.e., CG, CHG and CHH [28]. In *Arabidopsis*, the DNA methylation of CGs is maintained by methyltransferase 1 (MET1), a homolog of mammalian DNA-methyltransferase 1 (Dnmt1) [29], while plant specific chromomethylase 3 (CMT3) is required for the maintenance of CHGs [30]. Furthermore, all the methylation contexts, especially CHH methylation, are maintained by the *de novo* DNA methyltransferase Domains rearranged methyltransferase 2 (DRM2), the homolog of mammalian DNA-methyltransferase 3 (Dnmt3) [31]. In wheat, five putative DNA methyltransferases have been identified and classified into different categories based on their similarity to *Arabidopsis* or mammals [32]. DNA hypomethylation or hypermethylation can happen in response to various stresses, and thus regulate gene expression and subsequent plant physiology [27,33,34]. DNA methylation is also affected in response to Cd, arsenic (As) and nickel (Ni) in human and mouse [35,36]. Similarly in plants, DNA methylation is altered in white clover (*Trifolium repens* L.), industrial hemp (*Cannabis sativa* L.) plants, oil seed rape (*Brassica napus*) and radish (*Raphanus sativus* L.) in response to metal stress [37,38,39]. In *Posidonia oceanica*, Cd treatment induces the DNA hypermethylation and heterochromatinization [40]. In rice (*Oryza sativa*), DNA methylation levels were altered in response to Cd [41], and the progenies of rice plants that have been exposed to metal stress in their life cycle exhibited altered DNA methylation levels [42], indicating that DNA methylation plays an important role in plant response to metal stress. However, the link between DNA methylation and metal stress tolerance in crop plants, especially wheat, and the underlying epigenetic mechanism have not been investigated yet.

Here, we first screened several wheat-cultivated varieties against Pb toxicity. The Pirsabak 2004 and Fakhar-e-sarhad varieties that presented highest Pb resistance and sensitivity, respectively, were further characterized for their resistance against Cd and Zn toxicity. The resistance level was evaluated by measuring antioxidant activities and the accumulation of Pb, Cd and Zn in their roots. We hypothesized that the variation of resistance could be due to a different efficiency of metal detoxification such as subcellular sequestration or transportation. Therefore, we evaluated the expression level of the root-expressed *TaABCCs* and *TaHMA2* transporters in the presence of Pb, Cd or Zn in the resistant Pirsabak 2004 and sensitive Fakhar-e-sarhad varieties. To explore the underlying epigenetic mechanisms that regulate gene expression of transporters, we investigated the expression of DNA methyltransferases and quantified the DNA methylation levels at the promoter of the selected *TaABCCs* and *TaHMA2* metal transporters. 

## 2. Results

### 2.1. Genetic Diversity of Wheat Varieties Against Pb Toxicity

**Figure 1:**
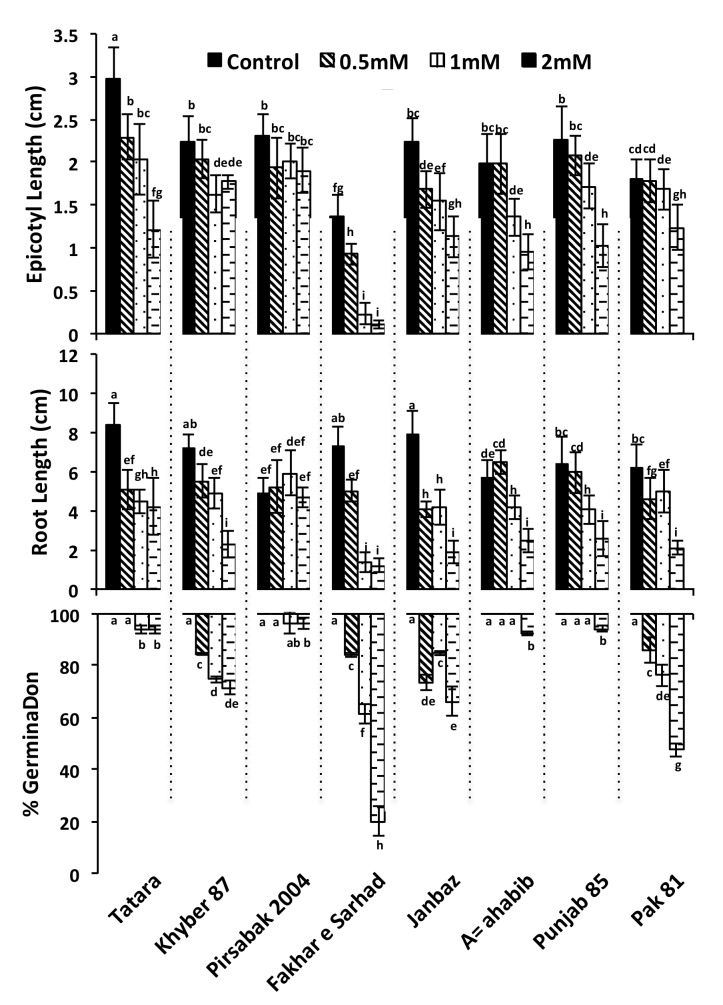
Screening of high-yielding wheat varieties based on their phenotypic characteristics against Pb toxicity. Seeds were grown on Murashige and Skoog (MS) media for 6 days under different concentrations of Pb(NO_3_)_2_. The results shown are the average of three biological replicates. Different letters indicate significant difference by a least significant difference (LSD) test (*p* ≤ 0.05). Error bars represent SD.

In order to investigate the metal toxicity mechanism in wheat, the high yielding wheat varieties were selected and screened against Pb toxicity to narrow down the genetic potential of each variety. The selected varieties were first screened for their germination capability in the presence of Pb (Figure 1). The tested varieties showed wide genetic diversity regarding Pb toxicity. Compared to the control, no effect on the germination rate was observed for Attahabib and Punjab 85 at 0.5 mM and 1 mM concentration of Pb(NO_3_)_2_, but a slight decrease in germination was observed at 2 mM. A higher sensitivity was observed for Fakhar-e-sarhad, Khyber 87, Janbaz and Pak 81, which showed a dose-dependent decrease in the germination rate. On the contrary, Pb(NO_3_)_2_ had no effect on the germination rate of Pirsabak 2004 at the tested concentrations. To further validate the genetic diversity observed in these varieties, we scored their primary root length and epicotyl length (Figure 1). Pb toxicity affected the primary root and epicotyl length in a dose-dependent manner in all the tested varieties, except Pirsabak 2004 whose growth was unaltered. Fakhar-e-sarhad showed a maximum decrease in the root length at 1 mM Pb(NO_3_)_2_ compared to control and no stronger effect was observed at 2 mM Pb(NO_3_)_2_. Similar to the root growth, the epicotyl growth was also severely impaired in Fakhar-e-sarhad at 1 mM and 2 mM Pb(NO_3_)_2_ compared to other varieties.

### 2.2. Pirsabak 2004 and Fakhar-e-sarhad Sensitivity to Pb, Cd and Zn 

**Figure 2:**
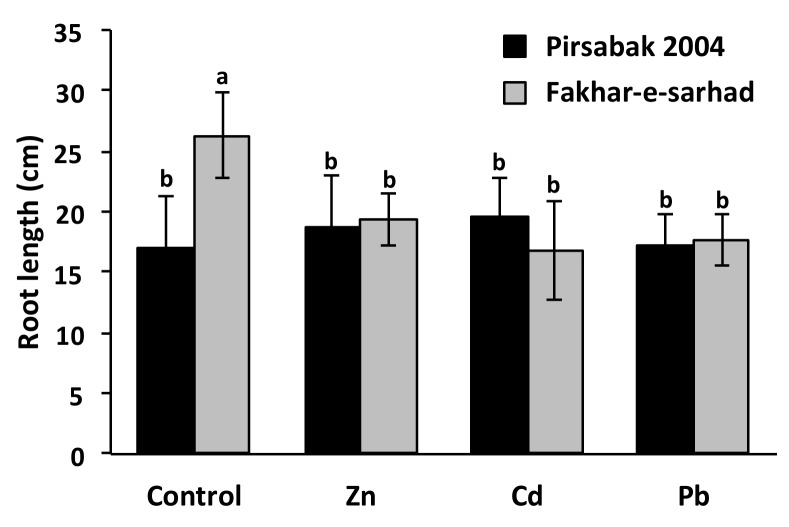
Pirsabak 2004 and Fakhar-e-sarhad varieties response to Cd and Zn stresses in hydroponic culture. The wheat seedlings were grown in hydroponic culture with 100 μM of Pb(NO_3_)_2_, ZnSO_4_ or CdCl_2_ and the root length was measured after two weeks of treatment. The results shown are the average of three biological replicates. Different letters indicate a significant difference by an LSD test (*p* ≤ 0.05). Error bars represent SD.

We further evaluated the resistant Pirsabak 2004 and sensitive Fakhar-e-sarhad varieties response to Pb treatment in hydroponics (Figure 2). In the hydroponic culture, 0.5 mM Pb was found toxic to seedlings, therefore, the heavy metal treatment was done by adding 100 µM of Pb(NO_3_)_2_. Fakhar-e-sarhad showed a decrease in root length in response to Pb toxicity in hydroponics, while the root length of Pirsabak 2004 did not change compared with control. This further confirms our result that Pirsabak 2004 is resistant to Pb toxicity. We hypothesized that Pb resistant varieties could also be resistant to other divalent ions, such as Cd and Zn. We therefore investigated the response of Pirsabak 2004 and Fakhar-e-sarhad varieties in hydroponic experiment supplemented with Cd or Zn (Figure 2). The results showed that the root length in Pirsabak 2004 did not change in response to Cd and Zn stresses, whereas the root length of Fakhar-e-sarhad decreased compared with control. This indicates that Pirsabak 2004 is also resistant to Cd and Zn stresses in hydroponic culture.

### 2.3. Antioxidant Activity in Pirsabak 2004 and Fakhar-e-sarhad in Response to Metal Stress 

**Figure 3:**
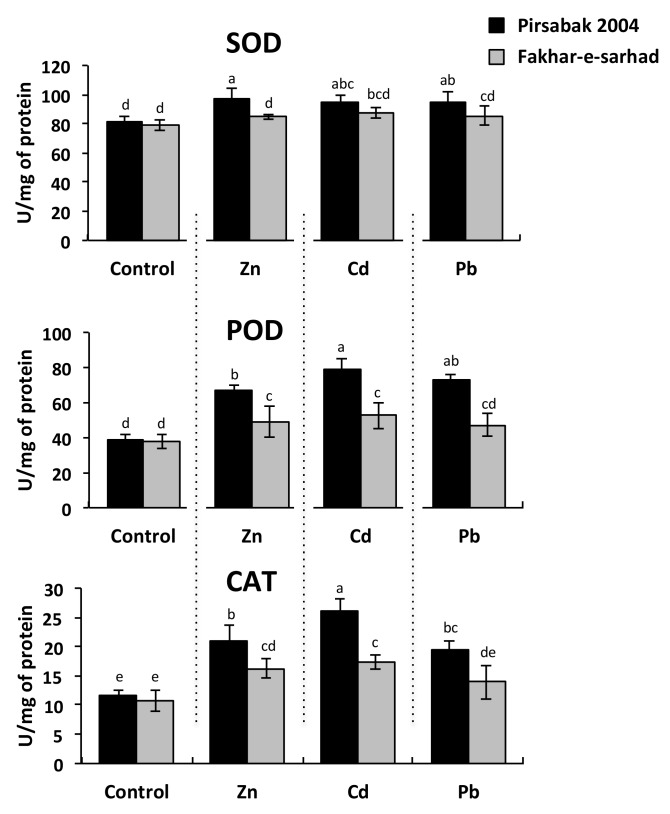
Superoxide dismutase (SOD), peroxidase (POD) or catalase (CAT) anti-oxidant levels in Pirsabak 2004 and Fakhar-e-sarhad varieties in response to 100 μM of Pb(NO_3_)_2_, ZnSO_4_ and CdCl_2_ in the hydroponic culture. The results shown are the average of three biological replicates. Different letters indicate a significant difference by an LSD test (*p* ≤ 0.05). Error bars represent SD.

The evaluation of a wheat response to metal toxicity can be achieved through the measurement of antioxidant activity of the superoxide dismutase (SOD), peroxidase (POD) or catalase (CAT). Pirsabak 2004 showed increased levels of SOD, POD and CAT activities in response to all the tested metals compared with control (Figure 3). Although Fakhar-e-sarhad showed slightly higher levels of SOD, POD and CAT than control, their levels were still significantly lower than Pirsabak 2004, indicating that the antioxidant activity is decreased in Fakhar-e-sarhad compared with Pirsabak 2004.

### 2.4. Accumulation of Pb, Cd and Zn in Pirsabak 2004 and Fakhar-e-Sarhad

**Figure 4:**
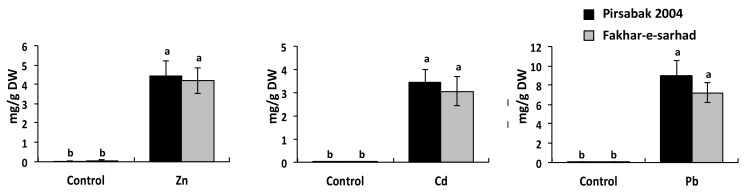
Pb/Cd/Zn accumulation in the roots of Pirsabak 2004 and Fakhar-e-sarhad varieties. The plants were grown in hydroponic culture with 100 μM of Pb(NO_3_)_2_, ZnSO_4_ or CdCl_2_, and metal accumulation was investigated after two weeks of treatment. The results shown are the average of three biological replicates. Different letters indicate a significant difference by an LSD test (*p* ≤ 0.05). Error bars represent SD.

We next investigated the accumulation of Pb, Cd and Zn in the roots of Fakhar-e-sarhad and Pirsabak 2004 varieties (Figure 4). Pirsabak 2004 and Fakhar-e-sarhad showed a similar amount of Pb, Cd and Zn in their roots. Moreover, Pb accumulation was higher than Cd and Zn in both varieties, which indicates that plants prefer to accumulate Pb compared with Zn and Cd. Pirsabak 2004 and Fakhar-e-sarhad showed non-significant amounts of Pb, Cd and Zn in shoots, indicating that both varieties do not differ in metal accumulation, and accumulated metals were mainly confined in roots. Together, these results indicate that the difference in toxicity was not due to a difference in metal accumulation.

### 2.5. Expression of Root Expressed TaABCCs and TaHMA2 transporters in Response to Pb, Cd and Zn Metal Stresses

**Figure 5:**
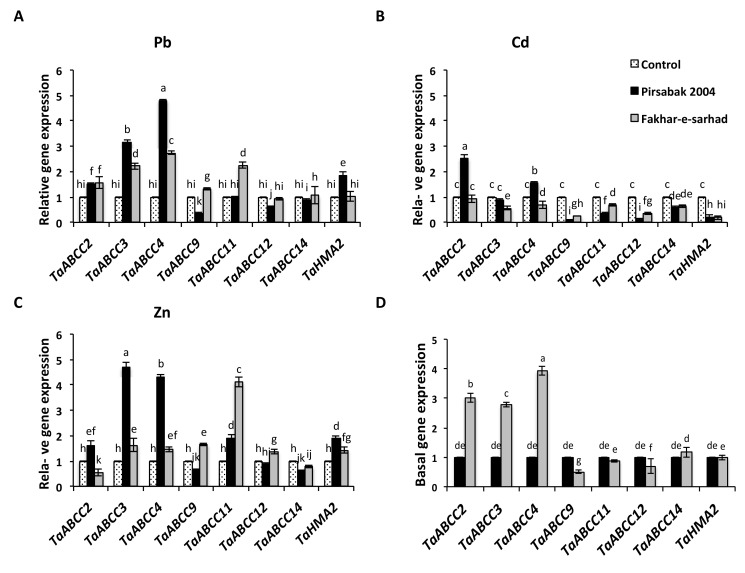
The expression of *TaABCCs/TaHMA2* transporters in response to Pb (**A**), Cd (**B**), Zn metals (**C**) and basal transcript levels (**D**) in the roots of Pirsabak 2004 and Fakhar-e-sarhad varieties. *18SrRNA* was used as an internal control. The results shown are the average of three biological replicates. Different letters indicate a significant difference by an LSD test (*p* ≤ 0.05). Error bars represent SD.

Since the resistant Pirsabak 2004 and sensitive Fakhar-e-sarhad varieties showed similar levels of metal accumulation in roots (Figure 4), we hypothesized that perhaps the resistant variety has transported the metals more efficiently in vacuoles compared to the sensitive variety. We quantified the expression of root expressed *TaABCC* transporters in both varieties (Figure 5). In general, Pb, Cd and Zn influenced the expression of several root expressed *TaABCC* genes, and all the tested genes responded differentially to each metal. The expression of *TaABCC2*, *TaABCC3* and *TaABCC4* was induced in both varieties (Pirsabak 2004 and Fakhar-e-sarhad) upon Pb treatment compared to their controls (Figure 5A), but Pirsabak 2004 showed a higher level of *TaABCC3* and *TaABCC4* transcripts than Fakhar-e-sarhad. Furthermore, *TaABCC9* and *TaABCC12* expression was down regulated in Pirsabak 2004 in response to Pb. However, the expression level of *TaABCC14* was largely unaltered by Pb treatment in both varieties. In response to Cd treatment (Figure 5B), the expression of *TaABCC2* and *TaABCC4* was induced only in Pirsabak 2004, whereas the expression of *TaABCC3* and *TaABCC4* was down regulated in Fakhar-e-sarhad compared to control. However, the expression of *TaABCC2* was not changed in Fakhar-e-sarhad compared to control in response to Cd treatment. Furthermore, the expression of *TaABCC9*, *TaABCC11*, *TaABCC12* and *TaABCC14* was down regulated in both varieties compared to the control in response to Cd treatment, but their expression levels were higher in Fakhar-e-sarhad than that of Pirsabak 2004. In response to Zn (Figure 5C), the expression of *TaABCC3*, *TaABCC4* and *TaABCC11* was increased in both varieties compared with control. However, *TaABCC3* and *TaABCC4* expression was much more induced in Pirsabak 2004 compared to Fakhar-e-sarhad, whereas the *TaABCC11* expression was more induced in Fakhar-e-sarhad compared with Pirsabak 2004. The expression of *TaABCC2* was increased in Pirsabak 2004 compared with control, whereas, the Fakhar-e-sarhad showed lower expression of *TaABCC2*. Furthermore, the expression of *TaABCC9* and *TaABCC12* was only induced in Fakhar-e-sarhad compared with control in response to Zn treatment. In addition, the expression of *TaABCC14* was down regulated in both varieties compared with control in response to Zn treatment. In brief, these results suggest that *TaABCC2*, *TaABCC3* and *TaABCC4* could contribute to Pb, Cd and Zn metal stress tolerance.

We also quantified the expression of *TaHMA2* in response to different metals in both varieties (Figure 5A–C). The *TaHMA2* expression in both varieties was increased in response to Zn compared with control. However, Pirsabak 2004 presented more induced expression compared with Fakhar-e-sarhad in response to Zn treatment (Figure 5C). In addition, *TaHMA2* expression was also increased in Pirsabak 2004 compared with control in response to Pb treatment, but did not change in Fakhar-e-sarhad (Figure 5A). The expression of *TaHMA2* was decreased in both varieties in response to Cd (Figure 5B). Thus, the expression of *TaHMA2* is specifically regulated in response to particular metals.

Since Pirsabak 2004 and Fakhar-e-sarhad showed different root lengths without any treatment, we wondered if this difference of phenotype could be to a differential basal expression of some genes due to their genetic background (Figure 5D). To our expectation, we found that the expression of *TaABCC2*, *TaABCC3* and *TaABCC4* was higher in Fakhar-e-sarhad compared with Pirsabak 2004 without any treatment, whereas, the expression of *TaABCC9* and *TaABCC12* was down regulated in Fakhar-e-sarhad compared with Pirsabak 2004, indicating that both varieties have different basal gene expression. However, the expression of *TaABCC11*, *TaABCC14* and *TaHMA2* did not show any remarkable difference between the two varieties.

### 2.6. DNA Methyltransferase Expression in Response to Pb, Cd and Zn Metal Stresses

**Figure 6:**
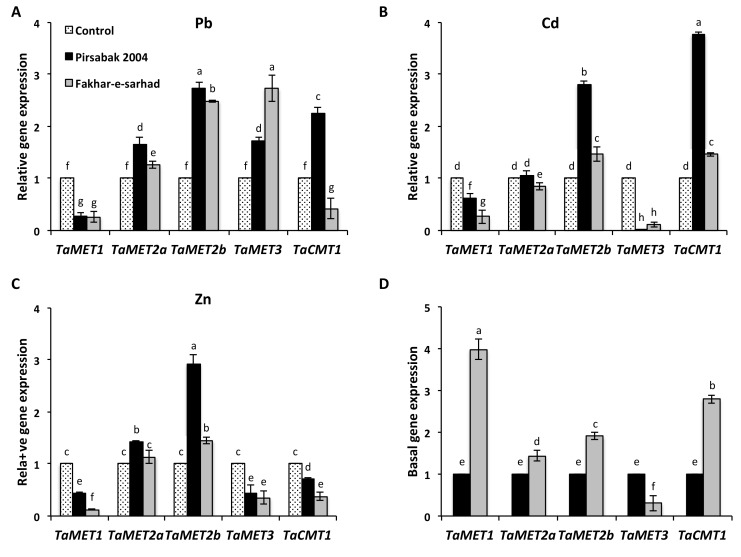
The expression of DNA methyltransferases in response to Pb (**A**), Cd (**B**), Zn metals (**C**), and their basal expression (**D**) in the roots of Pirsabak 2004 and Fakhar-e-sarhad varieties. *18SrRNA* was used as an internal control. The data presented are the average of three biological replicates. Different letters indicate a significant difference by an LSD test (*p* ≤ 0.05). Error bars represent SD.

We next investigated the expression of DNA methyltransferases, and our results showed their differential expression in response to Pb, Cd and Zn metal treatment (Figure 6). In response to Pb (Figure 6A), the expression of *TaMET1* was decreased in both Pirsabak 2004 and Fakhar-e-sarhad compared with control, whereas, the expression of *TaMET2a*, *TaMET2b* and *TaMET3* was increased in both varieties compared with control. However, the levels of *TaMET2a*, *TaMET2b* and *TaMET3* were different in Pirsabak 2004 and Fakhar-e-sarhad. The expression of *TaCMT1* was higher in Pirsabak 2004 compared with control in response to Pb treatment, whereas its expression was found lower in Fakhar-e-sarhad compared with control. In response to Cd treatment (Figure 6B), the expression of *TaMET2b* and *TaCMT1* was increased in Pirsabak 2004 and Fakhar-e-sarhad compared with control, but their expression was higher in Pirsabak 2004 compared with Fakhar-e-sarhad. On the contrary, the expression of *TaMET3* was strongly decreased in both Pirsabak 2004 and Fakhar-e-sarhad compared with control in response to Cd treatment. Similarly, the expression of *TaMET1* and *TaMET2a* was down regulated in Fakhar-e-sarhad compared with control in response to Cd treatment. In Pirsabak 2004, the *TaMET1* expression was also down regulated compared with control, but the levels in Pirsabak 2004 were higher than that of Fakhar-e-sarhad in response to Cd. However, the expression of *TaMET2a* did not change in Pirsabak 2004 in response to Cd treatment.

In response to Zn treatment (Figure 6C), the expression of *TaMET2b* was increased in both varieties compared with control, but Pirsabak 2004 levels were higher than that of Fakhar-e-sarhad. In contrast to these, *TaMET1*, *TaMET3* and *TaCMT1* expression was down regulated in response to Zn treatment in Pirsabak 2004 and Fakhar-e-sarhad. However, *TaMET1* and *TaCMT1* expression was higher in Pirsabak 2004 compared with Fakhar-e-sarhad in response to Zn treatment, indicating that the expression of a particular DNA methyltransferase is regulated depending on the metal stress and genetic background.

As we found that the expression of some *TaABCC* transporters was genetically different between these varieties without treatment (Figure 5D), we also compared the basal expression of methyltransferases in both varieties (Figure 6D). We found that the basal expression of *TaMET1*, *TaMET2a*, *TaMET2b* and *TaCMT1* was higher in Fakhar-e-sarhad compared with Pirsabak 2004, while the expression of *TaMET3* was lower in Fakhar-e-sarhad compared with Pirsabak 2004, which indicate that both varieties could have different DNA methylation levels and/or sites, thus could explain the difference in basal expression of some *TaABCC* transporters in Fakhar-e-sarhad and Pirsabak 2004.

### 2.7. DNA Hypomethylation of Pirsabak 2004 in Response to Pb, Cd and Zn Metal Stresses

**Figure 7:**
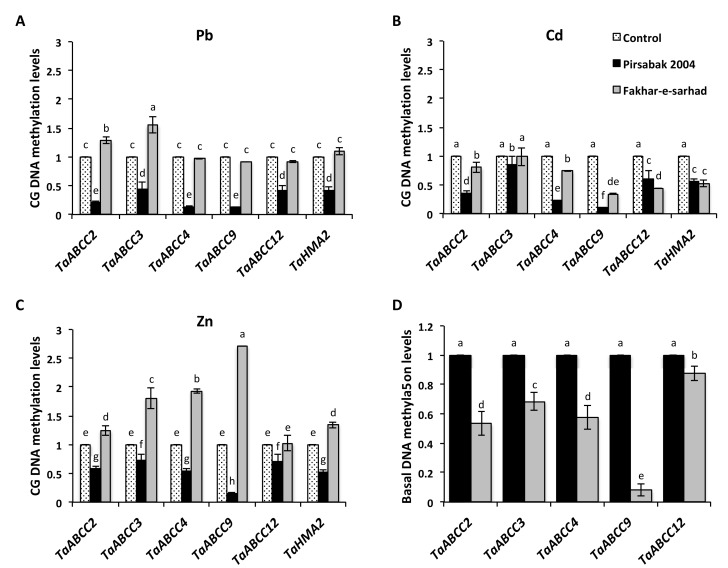
CG DNA methylation levels at the promoter of *TaABCC* transporters in response to Pb (**A**), Cd (**B**) and Zn metals (**C**) and the basal DNA methylation levels (**D**) in the roots of Pirsabak 2004 and Fakhar-e-sarhad varieties. DNA was digested with *AciI* and *hpaII* for CG DNA methylation. Equal amount of digested and undigested DNA were used as template for qPCR, % to non-digested DNA was calculated and relative to control is presented. The control of each variety was set to 1, therefore, presented only once in the graph. Basal DNA methylation represents the levels of DNA methylation of each variety in control conditions. The data presented are the average of three biological replicates. Different letters indicate a significant difference by an LSD test (*p* ≤ 0.05). Error bars represent SD.

We next quantified the DNA methylation levels at the promoter of *TaABCCs* and *TaHMA2* transporters in response to Pb, Cd and Zn treatments. DNA hypomethylation was observed at the promoters of the tested transporters in Pirsabak 2004 compared with control in response to Pb, Cd and Zn (Figure 7A–C, Figure S1). In response to Pb (Figure 7A), CG DNA methylation levels were reduced at the promoters of *TaABCC2*, *TaABCC3*, *TaABCC4*, *TaABCC9*, *TaABCC12* and *TaHMA2* in Pirsabak 2004 compared to the control. In Fakhar-e-sarhad, the DNA methylation levels were slightly higher at the promoter of *TaABCC2* and *TaABCC3* compared with control in response to Pb treatment. However, the CG DNA methylation levels did not change at *TaABCC4*, *TaABCC9*, *TaABCC12* and *TaHMA2* in Fakhar-e-sarhad compared with the control in response to Pb treatment. Similar to Pb, in response to Cd and Zn (Figure 7B,C), CG DNA methylations were also reduced at the promoters of all the tested *TaABCCs* and *TaHMA2* transporters in Pirsabak 2004 compared with control. Moreover, CG DNA methylation levels were decreased in response to Cd in Fakhar-e-sarhad, while the levels were generally increased in response to Zn compared with control. In general, DNA methylation levels in response to Pb, Cd and Zn were lower in Pirsabak 2004 compared with Fakhar-e-sarhad at the promoter of tested *TaABCCs* and *TaHMA2* transporters, thus probably explains the increase of *TaABCC2*, *TaABCC3, TaABCC4,* and *TaHMA2* expressions in Pirsabak 2004 in response to metal stress.

We also investigated whether the different basal expression of *TaABCCs* in both varieties is due to the different levels of DNA methylation on these genes. Basal DNA methylation levels were decreased at the promoter of *TaABCC2*, *TaABCC3*, *TaABCC4*, *TaABCC9*, *TaABCC12* and *TaHMA2* in Fakhar-e-sarhad compared with Pirsabak 2004 (Figure S1 and Figure 7D). Among the tested genes, only *TaABCC3* showed the CHH/CHG DNA methylation at his promoter. Fakhar-e-sarhad showed lower CHH/CHG DNA methylation compared with Pirsabak 2004 at the promoter of *TaABCC3* (Figure S2).

## 3. Discussion

Heavy metal toxicity for the environment, plants and human life has become a major global problem. Heavy metals do not easily degrade or volatilize, which leads to their accumulation in the soil over years. Among these heavy metals, Pb and Cd are the most harmful because they can enter into the food chain through the soil, thus imposing a serious threat not only to plants but also to humans and livestock [2,4,6]. In this particular scenario, screening high yielding plant varieties against metal toxicity and their adoption in plant breeding programs is essential. Therefore, we first screened eight high yielding wheat varieties for their phenotypic sensitivity to Pb toxicity at different doses. We found that Pb resistant Pirsabak 2004 and sensitive Fakhar-e-sarhad wheat varieties are also resistant or sensitive to Cd and Zn. Furthermore, Pb, Cd and Zn metal stresses induce DNA hypomethylation at the promoter of some selected *TaABCC* and *TaHMA2* metal detoxification transporters in Pirsabak 2004, which is correlated with their increased gene expression and metal resistant phenotype.

In order to reduce the Pb and Cd concentration in the soils, a lot of efforts have been made in previous years, including the use of hyper-accumulating plants [43]. However, due to low biomass, long remediation time and narrow biological adaptability, the usage of hyper-accumulating plants could not meet the demands of large-scale applications. Therefore, in parallel to hyper-accumulating plants, evaluating the genetic potential of crop plants against heavy metal toxicity could be a valuable choice. In this study we chose high yielding wheat varieties and evaluated their response to Pb toxicity. Seed germination and seedling growth are some of the most important and earlier physiological processes that are affected in wheat plants in response to metal stress [44]. Thus, the ability of a seed to germinate and the increase in seedling growth in the presence of metal stress would indicate the level of tolerance to metal stress. Our results showed that germination percentage, epicotyl length and root length were largely unaffected in Pirsabak 2004 in response to Pb stress, while these phenotypes were the most severely affected in Fakhar-e-sarhad among all the tested varieties (Figure 1). Furthermore, the root length of Pirsabak 2004 was also found not affected in response to Cd and Zn stresses, while Fakhar-e-sarhad root length was significantly affected (Figure 2). These results indicate that Pirsabak 2004 and Fakhar-e-sarhad were the most resistant and sensitive varieties, respectively. Since Pb, Cd and Zn affected the germination percentage and seedling growth in Fakhar-e-sarhad, we expected the low crop yield of Fakhar-e-sarhad in metal contaminated soils. While on the contrary, we expected the better performance of Pirsabak 2004 in metal contaminated soils. Thus we recommend farmers to cultivate Pirsabak 2004 in metal contaminated soils to ensure the better crop yield compared with all the tested varieties. 

Plants exposed to heavy metals generate reactive oxygen species (ROS) such as O_2_^−^ and OH^−^, which cause oxidative damage to the cellular structure and functions [45]. Therefore, plants have developed a complex antioxidant response, including the production of antioxidant enzymes, such as SOD, POD and CAT. SOD catalyzes the conversion of O_2_^−^ into molecular O_2_ and H_2_O_2_, and CAT and/or POD further detoxify the H_2_O_2_ [46,47]. This indicates that the levels of antioxidant activities would indicate the ability of the plant to cope with the metal stress by limiting the impact of ROS. The levels of SOD, POD and CAT were significantly increased in response to metal stress in Pirsabak 2004 and Fakhar-e-sarhad compared with control, but the levels of SOD, POD and CAT in Pirsabak 2004 were significantly higher than that of Fakhar-e-sarhad (Figure 3). This indicates that the antioxidant activities are decreased in Fakhar-e-sarhad in response to Pb, Cd and Zn, which may contribute to its sensitive phenotype.

The genetic diversity of plants has been extensively studied based on morphological and biochemical evaluation in the pre-genomic era, while DNA (or molecular) markers were studied in the post-genomic era [48]. Besides genetic variation, epigenetic modifications can create epialleles that can be inherited independently and epigenetic variations evolve more quickly [27,49]. Therefore, epigenetic variations could be used in plant breeding programs [27]. Our data showed the DNA hypomethylation at the promoter of *TaABCC* genes in Fakhar-e-sarhad compared with Pirsabak 2004 (Figure 7D) in the control samples, indicating the epigenetic variations between Pirsabak 2004 and Fakhar-e-sarhad. In addition, Pirsabak 2004 and Fakhar-e-sarhad also differed in basal transcriptional responses (Figure 5D and Figure 6D) and root length in the control samples (Figure 1). Especially, the expression of *TaABCC2, TaABCC3* and *TaABCC4* was higher in Fakhar-e-sarhad compared with Pirsabak 2004, which is consistent with DNA hypomethylation at their promoters in Fakhar-e-sarhad. Together, our results indicate the genetic and epigenetic diversity of Pirsabak 2004 and Fakhar-e-sarhad. However, further studies are required to explore their epigenetic diversity. 

DNA methylation events in response to a metal exposure have also been reported in *Vicia faba*, rape seedlings, and *Arabidopsis* [38,50,51]. *Trifolium repens* L. and *Cannabis sativa* L. plants have already been reported to have different basal DNA methylation levels in their roots [37]. Moreover, the Cd and Ni metal treatments induce DNA hypomethylation in both *Trifolium repens* L. and *Cannabis sativa* L. DNA methylation changes in response to Cd stress depends on the plants, e.g., in *Brassica napus*, *Trifolium repens* L. and *Cannabis sativa* L., the Cd induces the DNA hypomethylation [37,38,50], while in *Vicia faba*, Cd induces the DNA hypermethylation. Our results showed that Pirsabak 2004 presents CG DNA hypomethylation in response to Pb, Cd and Zn metal stresses at the promoter of *TaABCC2*, *TaABCC3*, *TaABCC4* and *TaHMA2* transporters (Figure 7), while Fakhar-e-sarhad showed hypermethylation considering their basal DNA methylation level, and increased DNA methylation levels compared with control, especially in the case of Zn. These observations suggest that DNA methylation plays an important role in the resistance mechanism of metal stress in Pirsabak 2004.

**Figure 8:**
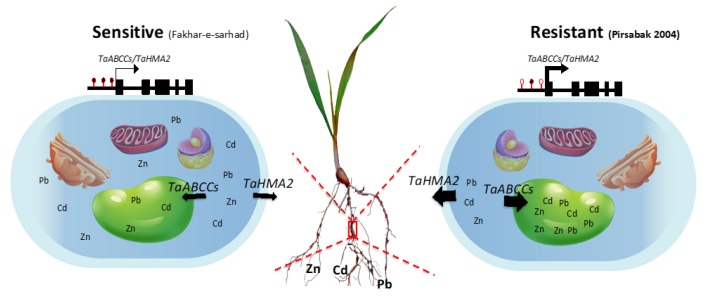
Proposed model for Pirsabak 2004 resistant and Fakhar-e-sarhad sensitive phenotypes. In response to metal stress, DNA methylation levels differently changed at the promoter of *TaABCCs* and *TaHMA2* transporters, and consequently changed their expression levels in Pirsabak 2004 and Fakhar-e-sarhad. Thus, vacuole sequestration of toxic metals through TaABCCs and export back to apoplast through TaHMA2 activity maintain the metal homeostasis and confer the resistant phenotype. Black filled circles represent DNA methylation, while open circles represent hypomethylation. The intensity of the arrow represents the expression level of genes.

Plants have evolved a system to prevent a cytotoxic concentration by effluxing the metals from the cytosol to the apoplast through the action of heavy metal ATPases (HMAs), also known as PIB-ATPases. The heavy metal ATPase2, TaHMA2, is a plasma membrane located transporter from wheat that was suggested to export the Zn/Cd toward the apoplast [24]. A yeast expressing wheat *TaHMA2* was found resistant to Zn/Cd and furthermore, the over expression of *TaHMA2* conferred a mild resistance against Zn and Cd in *Arabidopsis*, indicating the important function of TaHMA2 in metal tolerance in wheat. Interestingly, our results showed that the expression of *TaHMA2* was higher in Pirsabak 2004 compared with Fakhar-e-sarhad (Figure 5A–C) in response to Pb, Cd and Zn, which is consistent with DNA hypomethylation in Pirsabak 2004 compared with Fakhar-e-sarhad (Figure 7, Figure S1). This suggests that the increase in *TaHMA2* expression in response to metal stress likely contributes to the resistance in Pirsabak 2004. In parallel to HMAs, ABCC transporters have been reported to enhance resistance against metal stress in plants as well as in yeast by vacuole sequestration. Yeast Cadmium Factor 1 (YCF1), an ABCC transporter, has been reported to play an important role in metal tolerance in yeast [52,53]. Over-expression of *YCF1* in *Arabidopsis*, poplar and *Brassica* enhances the tolerance to Cd and Pb [54,55,56], suggesting the important function of ABCC transporters in metal detoxification. Furthermore, *Arabidopsis AtABCC1/AtABCC2* genes also play a role in conferring a resistance to Cd and mercury (Hg) stresses by vacuole sequestration [14]. Our results also showed the DNA hypomethylation (Figure 7, Figure S1) and increased expression of *TaABCC2*, *TaABCC3* and *TaABCC4* in Pirsabak 2004 compared with Fakhar-e-sarhad in response to Pb, Cd and Zn (Figure 5A–C). Notably, Pirsabak 2004 and Fakhar-e-sarhad accumulate similar amounts of Pb, Cd and Zn in their roots, which indicate that the resistance of Pirsabak 2004 is not due to less accumulation of toxic metals in the roots, but is likely due to the detoxification mechanism of plants. In this scenario, the enhanced activity of TaABCC2, TaABCC3, TaABCC4 and TaHMA2 transporters in Pirsabak 2004 likely contributes to the metal resistance of Pirsabak 2004. Therefore, we proposed that upon the exposure to Pb, Cd and Zn stresses, DNA hypomethylation occurred at the promoters of *TaABCCs* and *TaHMA2* in Pirsabak 2004, which will eventually lead to an increase in their transcription. The increased activity of TaABCCs may efficiently sequestrate accumulated Pb, Cd and Zn into the vacuole and in the meanwhile increased TaHMA2 activity may send the toxic metals back to the apoplast. The resulting metal concentration is not toxic to the cells, and meanwhile, the increased activity of SOD, POD and CAT scavenges the impact of ROS generated from metal toxicity. Together, these processes may lead to confer the resistance phenotype of Pirsabak 2004 (Figure 8). Furthermore, in response to Pb, Cd and Zn, TaABCCs mediated vacuole sequestration of toxic metals as well as export back of toxic metals to apoplast through the activity of TaHMA2 may not be sufficient in Fakhar-e-sarhad. The resulting metal concentration becomes higher in cells, and the antioxidant response may not be fully able to overcome the metal toxicity. Thus, relatively lower detoxification efficiency mediated by TaABCCs and TaHMA2, and decreased antioxidant activity in Fakhar-e-sarhad compared with Pirsabak 2004 may explain its sensitive phenotype. However, more functional studies of TaABCCs are required to validate this model in wheat.

In summary, our results demonstrated that the DNA methylation difference between resistant Pirsabak 2004 and sensitive Fakhar-e-sarhad varieties in response to Pb, Cd and Zn contributes to the metal tolerance through the regulation of the expression of metal detoxification transporters. This study highlights that the DNA methylation is an important parameter to confer heavy metal resistance in Pirsabak 2004. This study also recommends the cultivation of Pirsabak 2004 in metal contaminated soils. 

## 4. Materials and Methods

### 4.1. Plant Material

The selected high yielding varieties were collected from the seed stock of COMSATS University Islamabad, Abbottabad campus, Pakistan. The names of the selected varieties are Tatara, Khyber 87, Pirsabak 2004, Fakhar-e-sarhad, Janbaz, Attahabib, Punjab 85 and PAK 81. The pedigree detail of these varieties is given in Table S2.

### 4.2. Sowing and Growth Conditions for MS Media

The seeds were sterilized by dipping in 0.1% HgCl_2_ for 15–20 min, and then washed with double distilled water. Then the seeds were washed again in 70% ethanol for 10 min followed by four washes with distilled water. No Pb(NO_3_)_2_ as a control or different doses of Pb(NO_3_)_2_ i.e., 0.5 mM, 1 mM and 2 mM were added to Murashige and Skoog (MS) medium [57], supplemented with agar. Sterilized seeds of the selected varieties were sown on MS media in a growth chamber having 25 °C temperature and 16/8 h light/dark conditions. The data of morphological traits, root length, epicotyl length and germination percentage were scored after six days of sowing. The root and epicotyl lengths were measured from fifteen seedlings per replicate and the germination percentage was calculated from hundred seeds per replicate. All the experiments were performed in three biological replicates for control and treatments. The length of the primary root and the epicotyl length were measured by Image J, http://rsbweb.nih.gov/ij/.

### 4.3. Sowing and Growth Conditions for Hydroponics

The seeds were placed in a tray containing moist filter paper and placed in a growth chamber for 48 h. After germination, healthy seedlings (five seedlings per pot) having the same root length were wrapped in a foam layer and fixed in plastic cups, which were inserted into the plastic pots containing hydroponic solutions. The hydroponic solutions were composed of 0.2 mM KH_2_PO_4_, 1.0 mM K_2_SO_4_, 2.0 mM Ca(NO_3_)_2_, 2.0 mM CaCl_2_, 0.5 mM MgSO_4_ and 0.2 mM FeSO_4_.7H_2_O as a source of macronutrients while 5.0 µM H_3_BO_3_, 2.0 µM MnSO_4_, 0.5 µM ZnSO_4_, 0.3 µM CuSO_4_ and 0.01 µM (NH_4_)_2_Mo_7_O24 as micronutrients [58]. The nutrient solution was replaced twice in a week with freshly prepared solution of same strength. The heavy metal treatments were applied by adding 100 µM of CdCl_2_, Pb(NO_3_)_2_ and ZnSO_4_ in a hydroponic solution, while the control contained only hydroponic solution. All the experiments were performed in three biological replicates for control and treatments.

### 4.4. Atomic Absorption Analysis

After two weeks of the application of Pb(NO_3_)_2_, ZnSO_4_ and CdCl_2_ in the hydroponic culture medium plants were harvested. Root length was measured with measuring tape. In order to measure the uptake of Pb, Cd and Zn metals in root, dried roots were crushed and dried at 37 °C. The dried sample was ashed at 550 °C for 4–5 h in the furnace and allowed to cool down. Samples were digested for 3–4 h by adding 2 mL of 4 M HNO_3_. After 4 h, 8 mL of distilled water was added to make the final volume of 10 mL. Finally; the digested diluted plant material was filtered by using filter paper and analyzed for Pb, Cd and Zn on atomic absorption spectrophotometer (model, AAnalyst 700, PerkinElmer Inc., Shelton, CT, USA).

### 4.5. Extraction and Measurement of Antioxidant Enzymes 

Leaf samples were placed in liquid nitrogen immediately after their harvesting and stored at –80 °C until their analysis. The frozen leaf samples (0.5 g) were homogenized in 2.5 mL of 100 mM freshly prepared potassium phosphate buffer of pH 7 supplemented with 0.1 mM EDTA. Then the samples were centrifuged at 15,000 × g for 10 min at 4 °C and a supernatant was collected in eppendorf tubes, which were used for the analysis of antioxidants. Catalase (CAT), peroxidase (POD) and superoxide dismutase (SOD) activities were determined as described in [59]. The CAT activity was determined by monitoring the decomposition of H_2_O_2_ at 240 nm through spectrophotometer (U2020 IRMECO, Germany), while the activity of POD was measured by using guaiacol as substrate. The reaction mixture contained 0.1 M phosphate buffet of pH 7, 1% guaiacol, 0.4 M H_2_O_2_ and enzyme extract. Change in absorbance per unit time was measured at 470 nm. SOD activity was measured by photoreduction of nitroblue tetrazolium (NBT). Reaction mixture comprised 50 mM phosphate buffer of pH 7.8, 0.1 mM EDTA, 20 mM L-methionine, 750 µM NBT, 20 µM riboflavin and enzyme extract. The mixture was exposed to light for 15 min and absorbance was measured at 560 nm. The protein content in the leaves was measured by following the Bradford method [60]. The enzyme activity was expressed as U/mg of protein.

### 4.6. Gene Expression Analysis

Total RNA was extracted by using Trizol (Invitrogen, Waltham, MA, USA,) according to the manufacturer’s instructions from roots after 48 h of treatment with Pb, Cd and Zn. After DNase I treatment, reverse transcription was performed with Superscript III (Invitrogen) using the gene-specific primers. RT-qPCR was performed with the gene-specific primers using SYBR green Master Mix (Roche, Indianapolis, IN, USA) as described in [61]. The expression was corrected by using *18SrRNA* as an internal reference gene. The relative gene expression presented in Figure 5 and Figure 6 corresponds to the fold change of expression that was calculated by normalizing the expression of metal treated sample to the expression in the respective control sample of each variety. In order to clarify the fold change in response to a particular metal, the control of each variety was set to 1. The expression in control samples is referred as basal expression in the text. To compare the basal gene expression in Pirsabak 2004 and Fakhar-e-sarhad without any treatment, the expression levels of Pirsabak 2004 were set to 1 for each gene and then the expression in Fakhar-e-sarhad was normalized to the expression level of Pirsabak 2004. Primer sequences for gene expression are listed in Table S1.

### 4.7. DNA Methylation Chop-Quantitative PCR (Chop-qPCR)

Chop-PCR was performed as previously described by [62]. Briefly, genomic DNA was extracted with the CTAB method from wheat roots after 48 h of Pb, Cd and Zn treatments. Then the DNA was digested with *AciI* (R0551S New England Biolabs, Ipswich, MA, USA) and *hpaII* (R0171S New England Biolabs, Ipswich, MA, USA) for CG DNA methylation, and with *AluI* (New England Biolabs, Ipswich, MA, USA USA) and *haeIII* (R0108S New England Biolabs, Ipswich, MA, USA) for CHH/CHG methylation. Equal amount of digested and undigested DNA were used as template for qPCR, and normalized to undigested DNA. Basal DNA methylation represents the levels of DNA methylation of each variety in control conditions. To compare the basal gene DNA methylation levels in Pirsabak 2004 and Fakhar-e-sarhad, the DNA methylation levels of Pirsabak 2004 were set to 1 for each gene and then the DNA methylation levels in Fakhar-e-sarhad were normalized to Pirsabak 2004. For total DNA methylation levels, the *McrBC* enzyme (M0272S New England Biolabs, Ipswich, MA, USA USA) was used that specially digested methylated DNA, therefore, bands represent the non-DNA methylation levels. Chop-qPCR primers are listed in the Table S1.

### 4.8. Statistical Analysis

Experiments were conducted in a completely randomized design (CRD) with three replicates. The Shapiro–Wilk normality test was performed to test the normal distribution of data and the homogeneity of variance was tested by using the Levene’s test. After that analysis of variance (ANOVA) was performed followed by the least significant difference (LSD) test at *p* value ≤ 0.05 for each parameter. Statistical analyses were performed by using the Statistical Analysis System (SAS) software (SAS Institute Inc., Kerry, NC, USA) and the Statistical Package for Social Sciences (SPSS) software (version 11.0- SPSS Inc., Chicago, IL, USA).

## Supplementary Materials

Supplementary materials can be found at https://www.mdpi.com/1422-0067/20/19/4676/s1.
