# Supplementary material for: Lead, Cadmium and Zinc Phytotoxicity Alter DNA Methylation Levels to Confer Heavy Metal Tolerance in Wheat
Source: Int J Mol Sci. 2019 Sep 20;20(19):4676. doi: 10.3390/ijms20194676 (PMC6801570; doi:10.3390/ijms20194676)
Supplement: Supplementary file 1 [file ijms-20-04676-s001.pdf]

International Journal of *Molecular Sciences*

MDPI

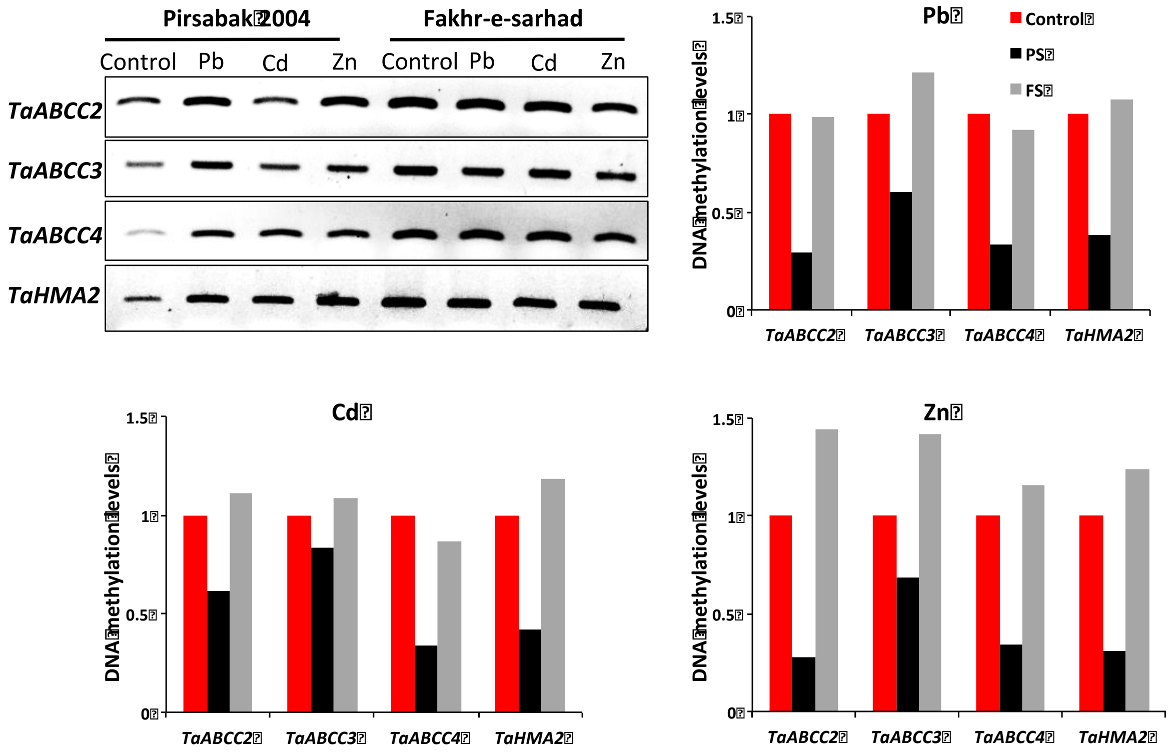

Figure 1. DNA methylation levels at the promoter of selected *TaABCCs*/*TaHMA2* transporters in Pirsabak 2004 (PS) and Fakhr-e-sarhad (FS). For total DNA methylation levels, McrBC was used that specially digested methylated DNA, therefore, bands represent the non-DNA methylation levels. Bands intensity was calculated with ImageJ for the bar graphs. The values from the control were divided by the values from the metal stress that represent the DNA methylation levels.

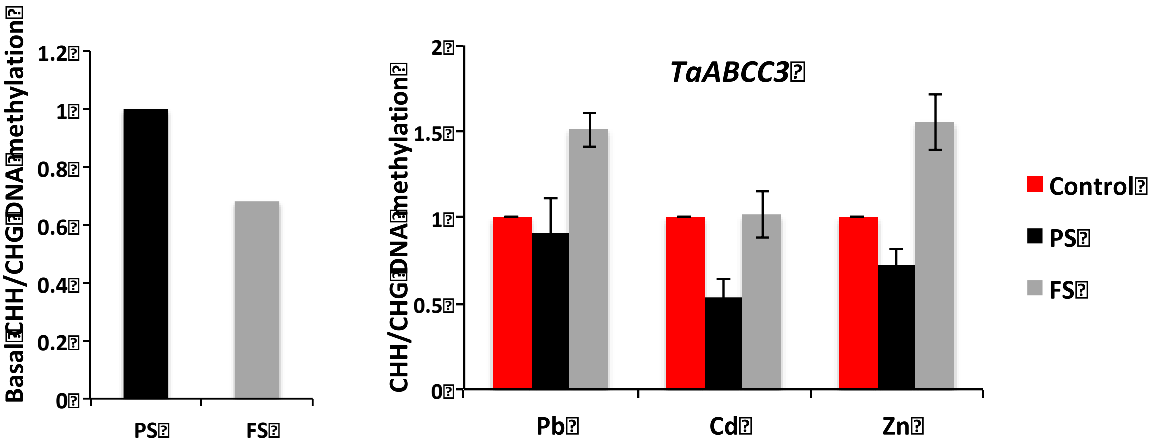

Figure 2. CHG/CHH DNA methylation levels at the promoter of *TaABCC3* transporter. Basal levels (A), and in response to Pb, Cd and Zn metals (B) in the roots of Pirsabak 2004 (PS) and Fakhr-e-sarhad (FS) varieties are presented. DNA was digested with AluI and haeIII for CHH/CHG methylation. Equal amount of digested and undigested DNA were used as template for pPCR, % to non-digested DNA was calculated and relative to control is presented.

Table 1. Primers used in this study.

Gene		Sequence 5'-3'
**qRT-PCR**		
TaMET3	F	ACGATAGCATATGTACTACAGCG
	R	GCGAGCACTCATCTTTGTTCTAC
TaCMT	F	TCCTCCGTTGGGACCCCT
	R	GACTAGACTCGAACCCCACAC
TaMET2b	F	TTAACCAATGCTTGACTGTGGTG
	R	AGCACCGTTTTGATTCAGGGTAT
TaMET1	F	AAGCTGCTGTGTTTGTGGATAAC
	R	TCAGCAAGTTTAGCTGCTTGTTC
TaMET2a	F	GGTGTCTGATACACATGACCAGT
	R	CCAAACAGAAGTAGCGTGGTCTA
TaHMA2	F	GGGCATCCGCTTATTTGG
	R	TTCCACTGCCTTTCTCCCTC
TaABCC2	F	GGGAGTGCATGATTTGCGGTGTAGG
	R	TA CCC TGG CCA AGC ACA CAA GTT GCC
TaABCC3	F	TTGCTAGCAATTACTGGATGGCGTG
	R	TGGAATCGAAGAAAGACATAGAGC
TaABCC4	F	CATTGCTAGCAATTACTGGATGGC
	R	GATGCGCCCACTTGGAGTGGAATC
TaABCC9	F	AGGTCTGGTGAAGGAATTCGATGC
	R	GAGGTTGGACGAGCGGTC
TaABCC11	F	CATTCTGTGTCGGGGCATGATAGA
	R	TGCATTTTGAGCTAGTTCTCTCCATC
TaABCC12	F	ATTGTCCATCTTCATCACAGTTGAAATGCGG
	R	CTTGATGAGTGCACAGCCAATGTTGACAATCA
TaABCC14	F	ACCACTCTCCTGAGCGCG
	R	AGAGGATCGACGTTGCTCCTTAC
Ta18SrRNA	F	GTGACGGGTGACGGAGAATT
	R	GACACTAATGCGCCCGGTAT
**ChOP-PCR**		
TaHMA2	F	TTGGGCTAACTAACCTTCTTCTC
	R	CATTAACTGGCAAACCCACCTA
TaABCC2	F	GTACATCCATGGGATTTGGTTGC
	R	TTGCTCACCATCACCAATACTGT
TaABCC3	F	CTCTGCTTCCGAGGAGCCTC
	R	CAAGGTCAGGGACGTCGTCG
TaABCC4	F	GAGCCTGCTCTCTTGCATTCTT
	R	CTTGCCGAACAGTATGTTCTCCTG
TaABCC9	F	CCGGGCACCCGGTGATCA
	R	ACGAACCTGTCCACGAGCGA
TaABCC12	F	TTAATTTTCCTCTGCATCGCACG
	R	TCAACCATGTATCCACTATAAGTGTCG

Table 2. Pedigree information of wheat varieties used in this study.

S. No	Varieties	Parenatge / Pedigree
1	Pak 81	KVZ//BUHO//KAL/BB<br>CM33027-F-15M-500Y-0M-76B-OY-OPAK
2	Fakhr-e-Sarhad	PFAU"S"/SERI/BOW "S"<br>CM85295-010-TOPY-2M-0Y-0M-3Y-0M
3	Tatara	JUP/ALO"S"//KLT"S"/3/VEE"S"<br>M79510-024Y-2M-05Y-01M-1Y-0B
4	Khyber 87	KVZ/TRM//PTM/ANA-CM 43930<br>CM 43903-H-4Y-1M-1Y-3M-2Y-0B
5	Pirsabak 2004	KAUZ/STAR
6	Janbaz	GEN*2//BUC/FILK/3/BUCHIN
7	Atta Habib	INQILAB 91*2/TUKURU<br>CGSS99B00015F-099Y-099M-099Y-31Y- OB
8	Punjab-85	KVZ/TRM//PTM/ANA<br>CM43903-H-4Y-1M-1Y-3M-3Y-OB-0PAK
